# Visualization of the role of host heme on the virulence of the heme auxotroph *Streptococcus agalactiae*

**DOI:** 10.1038/srep40435

**Published:** 2017-01-16

**Authors:** Laetitia Joubert, Jean-Baptiste Dagieu, Annabelle Fernandez, Aurélie Derré-Bobillot, Elise Borezée-Durant, Isabelle Fleurot, Alexandra Gruss, Delphine Lechardeur

**Affiliations:** 1 Micalis Institute, INRA, AgroParisTech, Université Paris-Saclay, 78350 Jouy en Josas, France; 2 Fromagerie BEL, 7 boulevard de l’Industrie, 41100 Vendôme, France; 3 Unité ISP, INRA, 37380 Nouzilly, France

## Abstract

Heme is essential for several cellular key functions but is also toxic. Whereas most bacterial pathogens utilize heme as a metabolic cofactor and iron source, the impact of host heme during bacterial infection remains elusive. The opportunist pathogen *Streptococcus agalactiae* does not synthesize heme but still uses it to activate a respiration metabolism. Concomitantly, heme toxicity is mainly controlled by the HrtBA efflux transporter. Here we investigate how *S. agalactiae* manages heme toxicity *versus* benefits in the living host. Using bioluminescent bacteria and heme-responsive reporters for *in vivo* imaging, we show that the capacity of *S. agalactiae* to overcome heme toxicity is required for successful infection, particularly in blood-rich organs. Host heme is simultaneously required, as visualized by a generalized infection defect of a respiration-negative mutant. In *S. agalactiae*, HrtBA expression responds to an intracellular heme signal *via* activation of the two-component system HssRS. A *hssRS* promoter-driven intracellular luminescent heme sensor was designed to identify host compartments that supply *S. agalactiae* with heme. *S. agalactiae* acquires heme in heart, kidneys, and liver, but not in the brain. We conclude that *S. agalactiae* response to heme is organ-dependent, and its efflux may be particularly relevant in late stages of infection.

Heme is ubiquitous in biological systems and an essential cofactor of several enzymes^1^. The importance of heme resides in the unique properties of its iron center, including the capacity to undergo electron transfer, perform acid-base reactions, and interact with various coordinating ligands^2^. Recent evidence emphasizes another role of heme, as a signalling molecule that regulates the function of key proteins implicated in several cellular processes^3^,^4^. Most bacteria carry the enzymatic machinery for endogenous heme synthesis and are also capable of acquiring environmental heme. Once internalized, heme is incorporated into bacterial proteins and/or degraded to release iron^3^,^4^. However, numerous bacteria, designated as heme-auxotrophs, lack some or all the enzymes needed for autosynthesis, but still require this molecule for their metabolism. The inability to synthesize heme means that these bacteria rely on environmental heme sources. One heme auxotroph is the Gram-positive opportunistic pathogen *Streptococcus agalactiae* (Group B streptococcus, GBS), a commensal inhabitant of the human gastrointestinal and female genitourinary tracts. Pregnant women who carry GBS asymptomatically can transmit the bacterium to their newborns during delivery, sometimes resulting in devastating neonatal infections including septicemia and meningitis^5^,^6^,^7^. GBS has emerged in the last 2 decades as a cause of invasive infections in adults with predisposing underlying diseases such as diabetes and cancer^8^,^9^.

Heme iron redox reactions may generate reactive oxygen species (ROS), which accelerate membrane peroxidation and damage to cellular proteins and DNA^10^. Several pathogens have evolved diverse and sophisticated strategies to outcompete heme sequestration by the host and fuel their heme requirements, they also need to balance their intracellular heme concentration below toxic levels^11^,^12^,^13^. Tolerance to heme in numerous Gram-positive bacteria involves a heme-regulated efflux pump (HrtBA; Heme-regulated transport), as identified in *Lactococcus lactis, Staphylococcus aureus, Bacillus anthracis*, and *Corynebacterium diphteriae*^14^,^15^,^16^. Pump-defective mutants are heme hypersensitive^3^,^15^,^17^. In *S. aureus* and *B. anthracis*, the HssRS (Hss; heme sensing system, R; regulator, S; sensor) two-component system (TCS) regulates HrtBA transporter expression^16^,^18^ in response to exogenous heme. Interestingly, *L. lactis* and other food/commensal heme auxotroph bacteria seem to have evolved a distinct response system that relies on intracellular heme sensing by the transcriptional regulator HrtR through heme binding^15^. In GBS, *hrtBA* orthologs were identified whose transcription is induced by hemin^19^. HssRS orthologs were also identified, but their exact function and regulation remain to be characterized. We previously proposed that HrtBA would protect cells in a heme-rich environment, e.g., during infection, when host red blood cells are degraded^19^. A distinct porphyrin efflux network called Pef, for porphyrin efflux, also contributes to PPIX and heme homeostasis; this system responds to low PPIX/heme concentrations compared to HrtBA^19^.

GBS depends on exogenous heme to activate a respiration chain that *in vitro* increases bacterial fitness and survival compared to fermentation metabolism, and is required for full virulence in a neonate rat model^20^. Menaquinones, which are also required, are provided from the diet or the gut and are present in blood in low amounts^20^,^21^. Heme captured from the environment activates the final acceptor complex of the GBS aerobic respiration chain, the cytochrome *bd* quinol oxidase (CydAB, encoded by *cydAB* genes)^11^,^21^,^22^,^23^,^24^. In *L. lactis* and GBS, respiration gene expression appears to be constitutive, with a slight increase late in growth^20^,^22^,^25^. This suggests that metabolism can switch efficiently from fermentation to respiration upon availability of the required cofactors in the local environment.

Passage in blood is a crucial step for colonization and pathogenesis of numerous bacteria^26^. However, the mechanisms involved in heme homeostasis and the availability and impact of host heme during infection are still unclear. As a heme auxotroph, GBS is particularly suited for such a study, as heme levels are fully controlled by exogenous heme pools. Here, we developed bacterial and heme biosensors combined with bioluminescence imaging to investigate GBS heme management *in vitro* and during infection. We demonstrate that HrtBA has a major impact on GBS survival in the host by preventing lethal heme overdose. On the other hand, heme acquisition activates respiration metabolism, which is crucial for GBS infection in a mouse septicemia model. Finally, based on the heme sensor, we show that GBS responses to host heme *in vivo* occur preferentially in specific organs, which contrasts with overall GBS distribution in the host. These experiments establish that GBS responses to exogenous heme are required for invasion and growth in the hostile host environment.

## Results

### Role of *hrtBA* in controlling GBS heme toxicity

**Figure 1:**
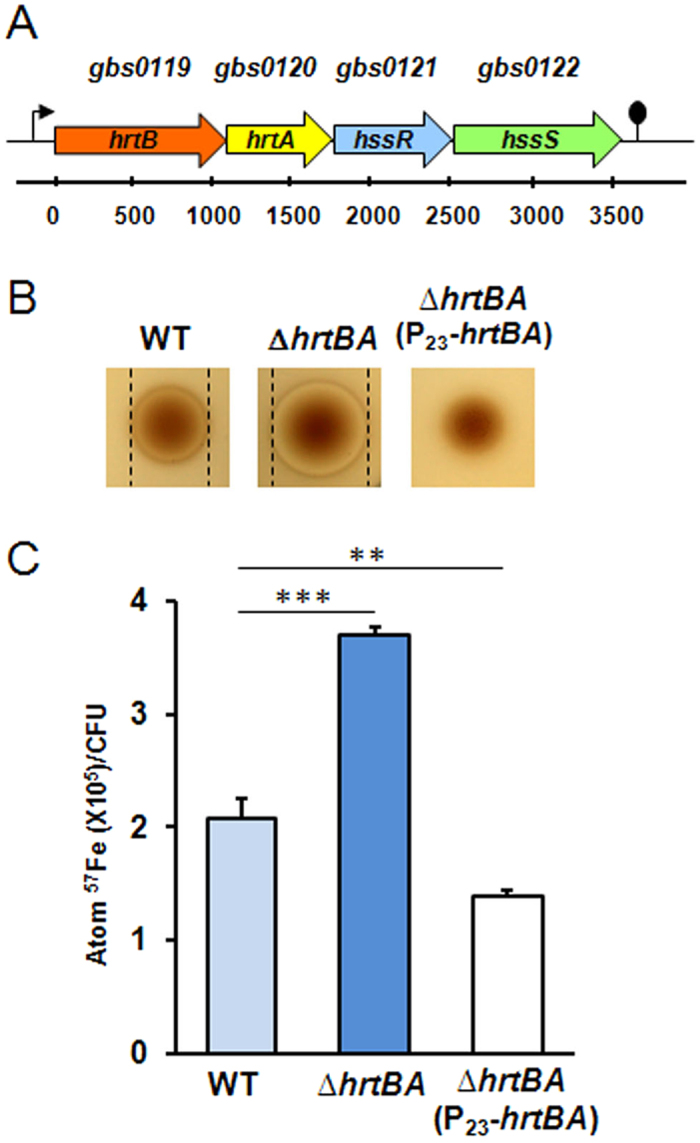
Heme toxicity regulation in GBS. (**A**) Operon organization of *hrtBAhssRS*. Schematic representation of the *hrtBAhssRS* operon in *S.agalactiae* (NEM316). *HrtB (gbs0119*) and *hrtA (gbs0120*) encode a permease and an ATPase, respectively while *hssS (gbs0122*) and *hssR (gbs0121*) encode the sensor histidine kinase and response regulator of a two-component system. (**B**) The *hrtBA* locus is involved in the control of heme toxicity in GBS. Stationary phase cultures of WT (NEM316), ∆*hrtBA* (NEMJJ8) and ∆*hrtBA* carrying a plasmid overexpressing *hrtBA* (P_23_-*hrtBA*) were plated in soft agar (Methods). Hemin (10 μl of a 10 mM stock solution) was pipetted directly onto plates, which were incubated at 37 °C for 24 h. Inhibition zones appear as a clearing in the center of each panel delimited by black lines. No inhibition zone was visible for ∆*hrtBA*(P_23_-*hrtBA*). **(C)** Heme cellular content is dependent on HrtBA expression. Heme content of GBS WT, ∆*hrtBA* and ∆*hrtBA*(P_23_-*hrtBA*) strains was assessed by ICP-MS on bacteria incubated with Fe^57^ labelled hemin (Methods). Results represent the mean ± standard deviation from triplicate samples and are representative of 3 independent experiments. Two-tailed Student test was used to determine P values: WT/Δ*hrtBA,* P = 0.0001; WT/Δ*hrtBA*(P_23_-*hrtBA*), P = 0.0036. **P < 0.01 and ***P < 0.001.

GBS0119 and GBS0120 showed respectively 30% and 45% amino acid identity with HrtB and HrtA of *S. aureus*^17^. In GBS, the operon comprises 2 additional genes, *gbs0121* and *gbs0122* (Fig. 1A and see below). GBS HrtBA was needed to manage environmental heme toxicity, as an in-frame Δ*hrtBA* deletion mutant (Table S1, strain NEMJ18, Supplementary Methods) exhibited hemin hypersensitivity compared to the WT (Fig. 1B; note that hemin refers to the oxidized molecule as present extracellularly, and heme refers to the reduced form of the molecule). The Δ*hrtBA* mutant was sensitive to hemin concentrations as low as 5 μM as determined in liquid culture assays (Fig. S1A,B). The role of HrtBA in managing heme toxicity was confirmed by complementation of the GBS Δ*hrtBA* strain expressing *hrtBA* from a constitutive promoter; growth of this complemented strain was insensitive to the same hemin concentration (Figs 1B and S1C, Table S1, Supplementary Methods).

To ascertain that increased hemin toxicity in the GBS Δ*hrtBA* mutant was linked to its accumulation, cellular ^57^Fe-labeled heme concentrations were quantified by inductively coupled plasma mass spectroscopy (ICP-MS)^15^. The ^57^Fe concentration was nearly 2-fold higher in Δ*hrtBA* than in the WT strain, while heme accumulation was reduced by 40% compared to the WT in the strain overexpressing HrtBA (Fig. 1C). These results confirm the functional link between HrtBA expression and heme accumulation in GBS. GBS HrtBA is thus a heme efflux permease that maintains cellular heme concentration by efflux to prevent toxicity.

### Control of host heme toxicity is critical for GBS virulence

Mammalian host organs are rich in blood but heme is mostly entrapped inside erythrocytes bound to hemoglobin or captured by hemoproteins, limiting its access^11^. We therefore asked whether *hrtBA*-mediated heme management is required during GBS infection in a mouse model. Mice were infected intravenously with WT GBS and the heme-sensitive Δ*hrtBA* strain, both carrying the p*lux* bioluminescent reporter plasmid, from which *Photorhabdus luminescens luxABCDE (lux*) genes are constitutively expressed^26^ (Table S1 and Supplementary Methods). A series of control experiments verified that luminescent GBS was suitable for *in vivo* study: i- the p*lux* plasmid remained stable in the absence of antibiotic pressure over a 24 h period, corresponding to 50 generations^27^ (Fig. S2A); ii- a linear correlation between colony forming units (CFU) and relative light units (RLU) was established *in vitro*, demonstrating that luminescence intensity correlated with CFU (Fig. S2B); iii- *lux* operon expression did not alter GBS growth rate (Fig. S2C). We conclude that progression of GBS infection can be followed by monitoring bioluminescence.

**Figure 2:**
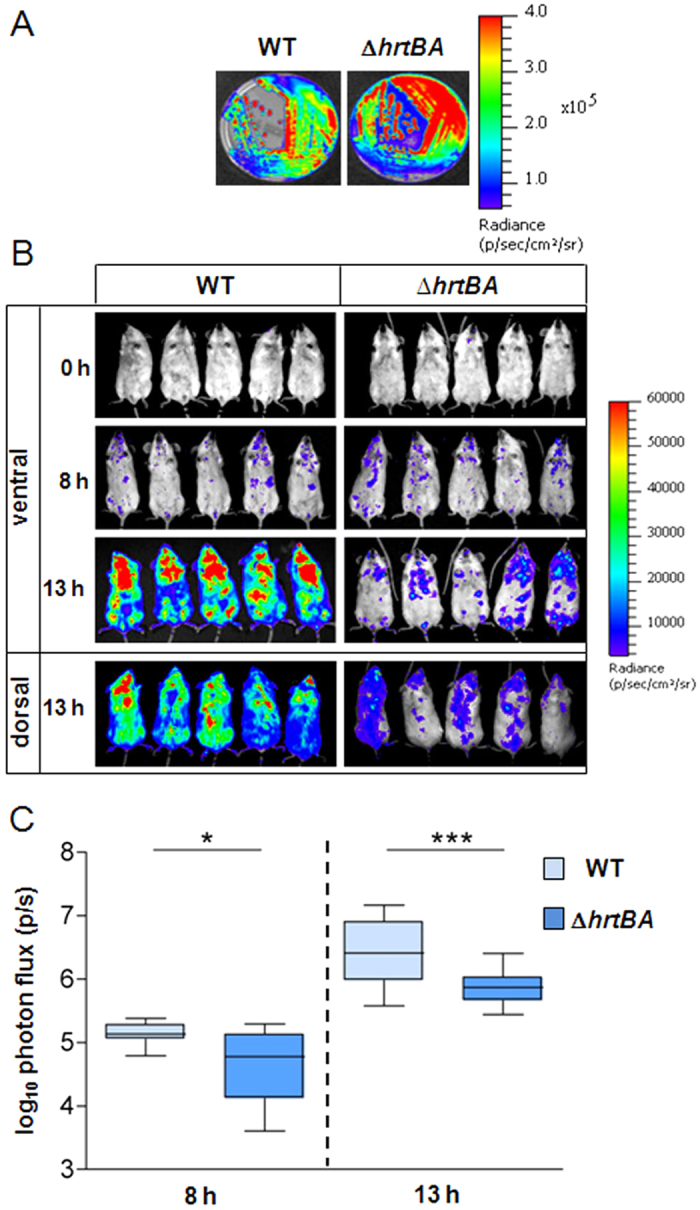
HrtBA expression is essential for full GBS virulence. (**A**) Luminescence of WT(p*lux*) and Δ*hrtBA*(p*lux*) streaked on solid agar plates. Luminescence was imaged with the IVIS 200 system (acquisition time, 1 min; binning 8) (Methods). (**B**) Course of infection of WT and Δ*hrtBA* GBS strains in mice. BALB/c mice were infected by intravenous injection of 2.10^7^ CFU of luminescent WT(p*lux*) and Δ*hrtBA*(p*lux*) GBS strains. Light emission in whole anesthetized animals was acquired and imaged in an IVIS 200 imaging system (acquisition time, 10 min; binning 8) immediately following bacterial inoculation (t = 0), at 8 h and 13 h (Methods). Batches of 3 mice for each time are shown and are representative of 3 independent experiments. (**C**) Quantification of bioluminescence in live mice at 8 h and 13 h post-infection with WT(p*lux*) and Δ*hrtBA*(p*lux*) as described in (**B**). Images were analyzed by measuring the total light flux (number of photons per second). Light emission from mice at 0 time point was subtracted. Box and whiskers plot of data from 5 experiments (corresponding to n = 15 mice per time point per strain). Quantified luminescence values correlate with bacterial counts in different organs (e.g., Fig. 3A). Two-tailed Mann–Whitney analyses was usedto determine P values: 8 h, P = 0.015; 13 h, P = 0.006. *P < 0.05 and ***P < 0.001 according to the Mann–Whitney test.

WT(p*lux*) and Δ*hrtBA*(p*lux*) strains exhibited a strong and similar autonomous luminescent signal as seen on agar plates (Fig. 2A) and in liquid cultures (Fig. S3A). Mice were infected with 2.10^7^ CFU of both strains by intraorbital injection (Fig. 2B). In mice infected with the WT(p*lux*) strain, the first clinical signs of infection (bristly hairs) were seen around 8 h post-injection, and overt signs of advanced disease (prostrated animals with spiky hairs) were seen 20–24 h post-injection. In parallel, whole animal luminescence following infection with GBS WT(p*lux*) was detectable in the first 8 h following the start of infection and then increased rapidly between 8 h and 13 h (Fig. 2B, left; Fig. 2C, WT). GBS WT(p*lux*) was detectable throughout mouse bodies (as seen on ventral and dorsal images), while higher levels of luminescence were localized in the head and thorax regions (Fig. 2B, WT at 13 h). At 8 h, luminescence observed in mice infected with Δ*hrtBA* and WT was comparable (Fig. 2B, right, and Fig. 2C, Δ*hrtBA*; note that the quantified number of photons in Fig. 2C gives a more accurate assessment of luminescence). However, luminescence intensity of Δ*hrtBA* was markedly lower at 13 h compared to the WT strain, indicating that infection progressed more slowly in the mutant. This observation is in line with the clinical status of the animals that showed less pronounced signs of disease.

**Figure 3:**
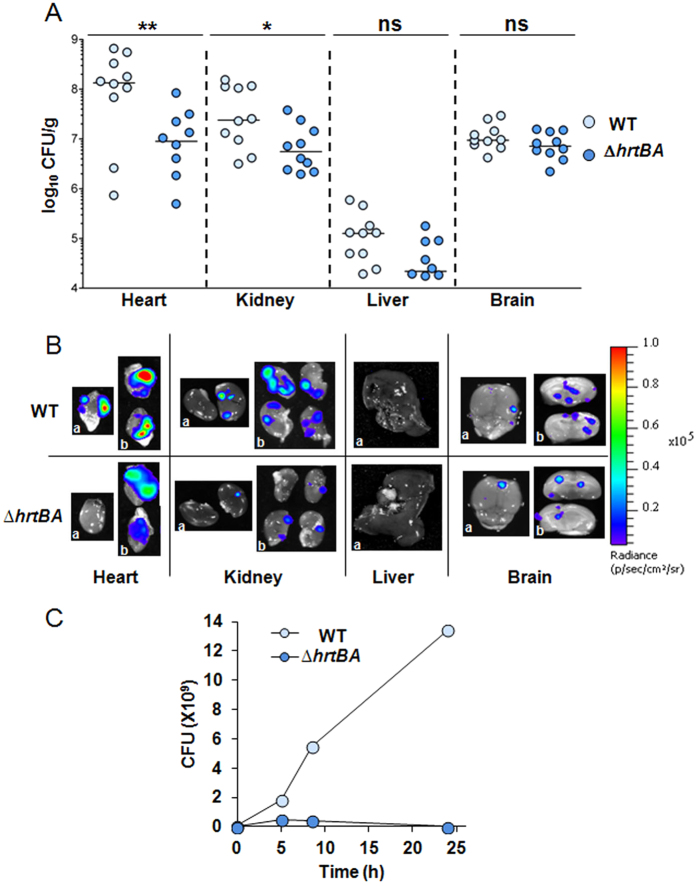
GBS depends on HrtBA expression to colonize host blood-rich organs. (**A**) Organ CFU of WT and Δ*hrtBA* GBS strains at 13 h post-infection. Mice infected as described in Fig. 2B were euthanized at 13 h post-infection. Dissected organs were homogenized and processed for CFU determinations (Methods). Symbols represent data from n = 8 to 10 mice from 3 independent experiments. Two tailed Mann–Whitney was used to determine P values: heart, P = 0.009; kidney, P = 0.029; liver P = 0.067; brain, P = 0.19. *P < 0.05; **P < 0.01; ***P < 0.001; ns, not significant (P > 0.05). (**B**) Bioluminescence from dissected organs. Indicated organs were imaged with the IVIS 200 (acquisition time, 5 min; binning 8). Representative organs from systematically infected mice sacrificed 13 h following the start of infection as in (**B**). a, top view; b, cross section of the indicated organs. **(C)** Growth of WT and Δ*hrtBA* in mouse blood. Bacteria from an ON preculture were diluted to an OD = 0.01 in 1 ml of freshly collected (<24 h) and heparinized blood of 6 weeks old BALB/c mice (Janvier, Le Genest Saint-Isle, France). At the indicated times, 100 μl of the culture was serially diluted and plated on agar plates for determination of the CFU. Results are representative of three independent experiments.

Infection of different organs by the WT and Δ*hrtBA* strains was compared. Mice were euthanized 13 h post-infection. Bacteria were enumerated in heart, kidney, liver and brain (Fig. 3A) after luminescence in dissected organs was monitored (Fig. 3B). The highest levels of WT GBS were recovered from the heart and kidneys (Fig. 3A, WT) and correlated to strongly luminescent localized spots on the dissected tissues (Fig. 3B (heart and kidney, WT)). Lower bacterial counts (3 logs compared to the heart) were enumerated from livers, in keeping with the absence of luminescence in this organ (Fig. 3B, liver) and indicating the detection limit of this sensor system. GBS colonization of brain tissue (Figs 2B and 3A) was observed as discrete spots of luminescence on the surface and inside the organ, in accordance with its capacity to penetrate cerebral tissue (Fig. 3B). Bacterial burden was approximately 1 log lower in the heart, kidneys and liver upon infection with the Δ*hrtBA* strain (Fig. 3A), and paralleled the lower luminescence of infected organs (Fig. 3B). Interestingly, Δ*hrtBA* and WT strains colonized the brain to comparable levels (Fig. 3A,B), supporting the idea that heme challenge encountered by GBS in the brain is limited. Bacterial counts in lung and blood samples were too low for significant comparison between WT and Δ*hrtBA* strains. However, growth of Δ*hrtBA* and WT strains in fresh mouse blood revealed that Δ*hrtBA* failed to multiply in whole blood (Fig. 3C) highlighting the importance of HrtBA during prolonged exposure of GBS to blood.

We conclude that GBS might encounter toxic levels of heme in blood and blood-rich organs such as heart, kidney and liver during systemic infection in the mouse model, which necessitates expression of the heme efflux transporter HrtBA. The HrtBA requirement appears to be more pronounced late in infection. In contrast, GBS colonization of the brain was not altered for the Δ*hrtBA* strain, in keeping with the strict control of blood exchanges with the cerebral tissue.

### GBS virulence relies on host heme-activated respiration metabolism

**Figure 4:**
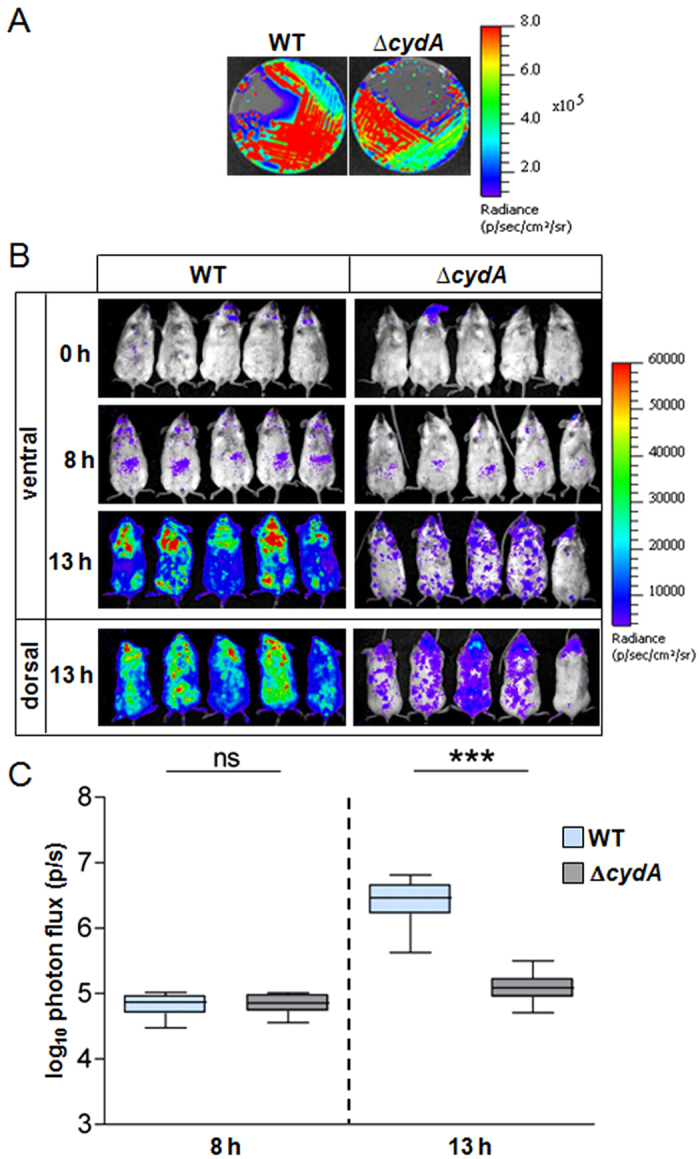
GBS virulence relies on host heme-activated respiration metabolism. (**A**) Bioluminescence of WT(NEM316) and Δ*cydA*(NEMJ17) transformed with p*lux* on agar plates. Luminescence was imaged with the IVIS 200 system (acquisition time, 1 min; binning 8) (Methods). (**B**) Course of infection of WT and Δ*cydA* GBS strains in mice. BALB/c mice were infected by intravenous injection of 2.10^7^ CFU of luminescent WT(p*lux*) and Δ*cydA*(p*lux*) GBS strains. Animal luminescence was imaged as in Fig. 3A at the indicated time points by IVIS 200 (acquisition time, 10 min; binning 8). Five mice for each time are shown and are representative of 3 independent experiments. (**C**) Quantification of bioluminescence in live mice at 8 h and 13 h post-infection with WT and Δ*cydA* strains. Luminescence from live animals infected with WT(p*lux*) and Δ*cydA*(p*lux*) was determined as in (**B**). Box and whiskers plot of data from 3 experiments (corresponding to a total of n = 15 mice per time point). Two-tailed Mann–Whitney was used for data analyses: 8 h, P = 0.912; 13 h, P = 0.0001. ns, not significant (P > 0.05); ***P < 0.001.

The use of heme as a quinol oxidase cofactor leads to more robust GBS growth *via* respiration^20^,^21^.We explored the role of respiration metabolism as a main heme-requiring function in GBS infection using the p*lux* biosensor. The capacity of a Δ*cydA* (cytochrome A subunit) mutant that is respiration-defective transformed with p*lux*, Δ*cydA*(p*lux*) mutant strain (Table S1, NEMJ17, Supplementary Methods) to grow and sustain a successful infection in mice was tested as in Fig. 2. The Δ*cydA*(p*lux*) mutant emitted light similarly to the WT *in vitro* (Fig. 4A and S3B). The course of systemic infection by the Δ*cydA* strain was dramatically limited compared to the WT as seen by the biophotonic images of representative infected mice at 13 h post-injection (Fig. 4B). While results with both strains were similar at the 8 h time point (Fig. 4B, 8 h), mice infected with the Δ*cydA* mutant showed no sign of disease at 13 h post-injection while those infected with the WT were prostrated with spiky hairs. Consistently, quantification of total luminescence of injected mice showed a quasi-arrest in Δ*cydA* luminescence between 8 h and 13 h of infection, while infection by the WT strain progressed rapidly (Fig. 4C). The Δ*cydA* bacteria appeared to be disseminated in mice, while WT bacteria were concentrated preferentially in the thorax and head (Fig. 4B, 13 h).

**Figure 5:**
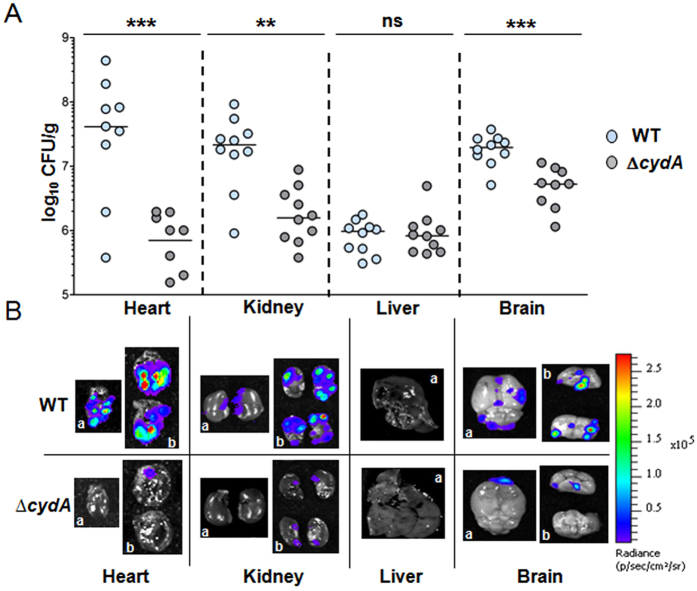
GBS capacity to colonize host organs requires heme-activated respiration metabolism. (**A**) Organ CFU of WT and Δ*cydA* GBS strains at 13 h post-infection. Mice infected as described in Fig. 2B were euthanized at 13 h post-infection and bacterial burden in dissected organs determined as in (**A**). Symbols represent data from n = 8 to 10 mice from 3 independent experiments. Two-tailed Mann–Whitney was used to determine P values: heart, P = 0.001; kidney, P = 0.0015; liver, P = 0.97; brain, P = 0.0004. *P < 0.05; **P < 0.01; ***P < 0.001; ns, not significant (P > 0.05). (**B**) WT(p*lux*) and Δ*cydA*(p*lux*) bioluminescence in dissected organs. Representative organs from infected mice were sacrificed 13 h post-infection. Organs were visualized in an IVIS 200 system (acquisition time, 5 min; binning 8).

Bacterial distribution in dissected organs was quantified (Fig. 5A). Compared to the WT, the Δ*cydA* bacterial load was 2 to 3 logs lower in heart and kidney while CFU levels in the liver were similar for both strains (Fig. 5A). CFU corresponding to Δ*cydA* strain in the brain were decreased by 1 log (Fig. 5A). In accordance with CFU results, representative organs of mice injected with the Δ*cydA* mutant showed little luminescence compared to the WT, as clearly observed in the heart, kidney and brain (Fig. 5B).

We conclude that while GBS must adapt to host heme toxicity by strictly limiting its intracellular accumulation (*via* HrtBA), it also needs to find heme within the host, particularly late in infection, to fulfill its needs for respiration metabolism. These results highlight the importance of heme management and acquisition during GBS infection.

### Internalized heme activates HrtBA expression *via* HssRS signaling

**Figure 6:**
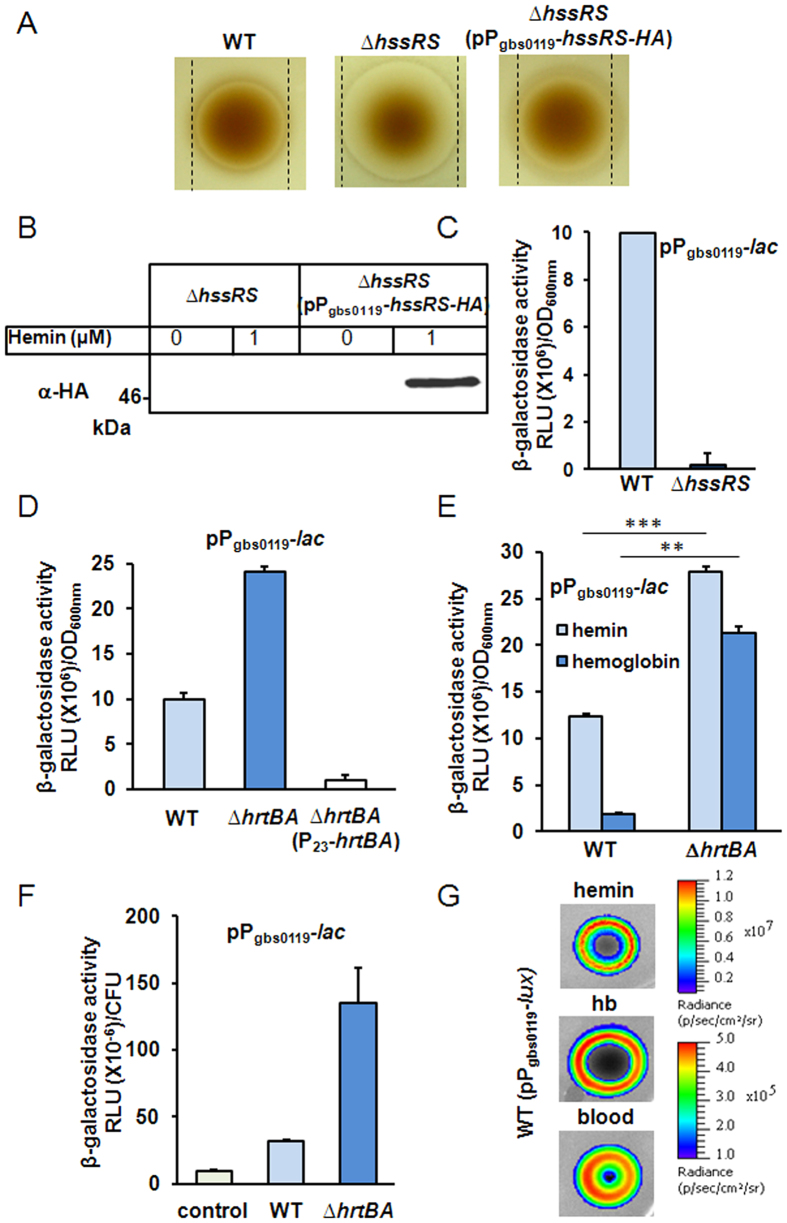
Intracellular heme controls the expression of *hrtBAhssRS* in GBS. (**A**) HrtBA expression is mediated by HssRS. Cultures of WT, ∆*hssRS* and ∆*hssRS*(pP_gbs0119_-*hssRS-HA*) GBS strains were plated as in Fig. 1B. (**B**) HssS is expression is induced by hemin. GBS ∆*hssRS* and ∆*hssRS*(pP_gbs0119_-*hssRS-HA*) strains were grown to OD_600nm_ = 0.5 and treated for 1 h with hemin as indicated. Western blot was performed with cell lysates using an anti-HA antibody (Supplementary methods). (**C**) The *hrtBAhssRS* operon is controlled by HssRS. β-galactosidase activity of WT and ∆*hssRS* GBS strains transformed with P_gbs0119_*-lac* was performed as in (**B**). (**D**) P_gbs0119_ induction depends on heme intracellular accumulation. WT, ∆*hrtBA* and ∆*hrtBA*(P_23_*-hrtBA*) carrying the P_gbs0119_*-lac* expression cassette were treated with hemin as in (**B**). (**E**) GBS internalizes heme from Hb. WT(pP_gbs0119_*-lac*) and ∆*hrtBA*(pP_gbs0119_*-lac)* GBS strains were incubated with 1 μM hemin or freshly prepared bovine Hb (Methods). β-gal expression was followed as described in (B). The Hb solution did not contain measurable amounts of free heme as verified by UV-visible spectroscopy. Two-tailed Student test was used to determine P values: WT (hemin/Hb), P = 0.0001; Δ*hrtBA* (hemin/Hb), P = 0.009. **P < 0.01 and ***P < 0.001. (**F**) GBS scavenges heme from blood. WT(pP_gbs0119_*-lac*), ∆*hrtBA*(pP_gbs0119_*-lac*), and negative control WT (pTCV-*lac*) were grown to OD_600nm_ = 0.5 and mixed with 25% BALB/c fresh blood for 3 h at 37 °C (Methods). Luminescence was determined as in (C) and CFU from blood samples were determined by serial dilutions on plates. (**G**) Heme dependent light emission by the heme sensing GBS strain. GBS WT(pP_gbs0119_-*lux*) was resuspended in soft agar and overlayed on agar plates. Hemin, Hb (10 μl of a 10 mM stock solution) or fresh BALB/c mice blood (10 μl) were directly spotted on plates. Plates were incubated at 37 °C for 24 h and luminescence was visualized using an IVIS 200 luminescence imaging system (Methods).

A bioinformatic search for potential activators of HrtBA expression identified *gbs0121* and *gbs0122* as encoding a regulator and sensor histidine kinase (HK) of a two-component system (Fig. 1A). These proteins share respectively 39% and 31% identity with the heme sensing system proteins HssR (heme sensing system regulator) and HssS (heme sensing system sensor) of *S. aureus*^17^,^18^. For both bacteria, the coding genes are adjacent to *hrtBA*. We tentatively renamed GBS0121 as HssR and GBS0122 as HssS. To verify the role of HssRS in HrtBA regulation, an in-frame deletion of *hssR* and *hssS* was constructed (Table S1, strain NEMJ19, Supplementary methods). The Δ*hssRS* GBS strain was hypersensitive to hemin as seen on agar plates (Fig. 6A) and liquid cultures (Fig. S3A,B). Complementation of the mutant Δ*hssRS* strain with *hssRS-HA* controlled by the P_gbs0119_ promoter (pP_gbs0119_-*hssRS-HA*, Table S1, Supplementary methods) further suggested that HssRS expression was required for HrtBA function (Fig. 6A, right panel; Fig. S4C). Finally, heme dependent expression of HssS tagged at its Ct with the HA epitope from Δ*hssRS*(pP_gbs0119_-*hssRS-HA*) was verified on Western blot (Fig. 6B). The heme-inducible promoter of the *hrtBAhssSR* operon was also fused to *lacZ* (P_gbs0119_*-lac*)^19^ (Table S1). In the WT strain, induction is linear in the range of 0.1 μM and 1 μM heme (below hemin toxicity concentrations in GBS, Figs S1A and S4A). In contrast, P_gbs0119_*-lac* expression in the Δ*hssRS* strain remained virtually null in the presence of 1 μM hemin compared to the WT strain (Fig. 6C) indicating a role of HssRS in transcriptional *hrtBAhssRS* activation. We conclude that the *hrtBAhssRS* operon is induced by heme *via* HssRS activation.

In *S. aureus*, heme-triggered HssRS activation of *hrtBA* expression involves HssS amino acid residues predicted to be localized in the extracellular domain (ECD, comprising amino acids 33–164)^18^,^28^,^29^ (Fig. S5). Surprisingly, GBS HssS lacks nearly the entire predicted heme sensing ECD as identified in *S. aureus* (Fig. S5), leading us to question the role of extracellular heme for GBS *hrtBAhssRS* induction. We hypothesized that intracellular, rather than extracellular heme, might be required to activate GBS *hrtBAhssRS* expression. To test this, we depleted intracellular heme levels by constitutively expressing the HrtBA heme efflux pump. P_gbs0119_*-lac* induction in response to 1 μM hemin was tested in strains that either accumulate intracellular heme (Δ*hrtBA*), or efflux heme (HrtBA overproducer) (Fig. 6D). Compared to the WT, P_gbs0119_*-lac* expression was about 2.5 times higher in Δ*hrtBA*. Importantly, expression was essentially non-detectable in the HrtBA overexpression strain, despite the presence of heme in the extracellular medium (Fig. 6D). Thus, β-gal expression correlated to heme cellular, and not extracellular, accumulation (also see Fig. 1C). Finally, this conclusion is consistent with β-gal expression of the *L. lactis* intracellular heme sensor P_hrt_*hrtR-lac* as a reporter of cytoplasmic heme^15^ in GBS WT and Δ*hrtBA* strains (Table S1 and Fig. S6). They further suggest that heme uptake and internalization are required for *hrtBAhssRS* induction, and that P_gbs0119_ functions as a heme sensor that detects and responds to heme bacterial accumulation.

Hemoglobin (Hb) and blood are physiologically available heme sources. To get insight into the capacity of GBS to internalize heme from Hb, we compared P_gbs0119_*-lac* induction in the presence of equivalent concentrations (1 μM) of hemin and Hb (Fig. 6E). β-gal expression was induced by Hb, although with lower efficacy than by free hemin. This result highlights the need for GBS to recover heme bound to Hb before its internalization. As expected, the Δ*hrtBA* mutant accumulated more heme from Hb than its WT counterpart (Fig. 6E). Similarly, fresh heparinized blood from BALB/c mice activated P_gbs0119_*-lac* at increased levels in the Δ*hrtBA* mutant (Fig. 6F). Altogether, these data show that GBS derives its intracellular heme from common host sources, and regulates its intracellular heme concentration *via hrtBAhssRS*.

We exploited the P_gbs0119_-*lac* heme sensor and Δ*hrtBA* mutants to evaluate GBS behavior in response to heme availability in the living host. For this purpose, we generated pP_gbs0119_-*lux* for *in vivo* use as a heme sensor (Table S1). As expected, the strain emitted light specifically in the presence of hemin, Hb and blood (Figs 6G and S4E).

### Heme sensing during systemic infection

**Figure 7:**
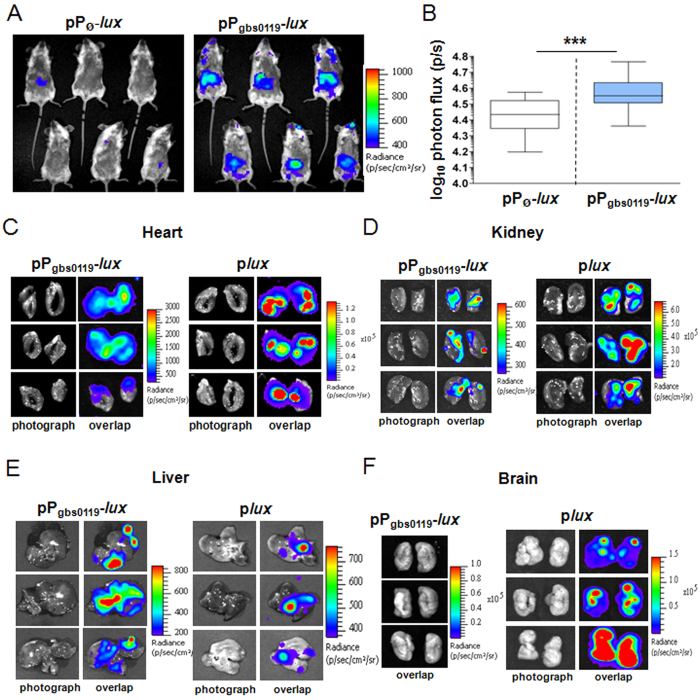
Heme sensing by GBS during systemic infection. (**A**) Heme sensing by GBS over the course of infection. Female BALB/c mice were infected with 2.10^7^ CFU of WT(pP_gbs0119_-*lux*) or WT(pP_Ø_-*lux*) strains. At 20 h post-infection, anesthetized mice were shaven and imaged organs in the IVIS 200 system (acquisition time, 20 min; binning 16; Methods). (**B**) Quantification of bioluminescence in live mice at 20 h post-infection. Luminescence of the abdomen of mice infected as in Fig. 5B was determined (Methods). Box and whiskers plot of data collected from 4 experiments (corresponding to a total of n = 25 mice per strain). The P value determined using a two-tailed Mann–Whitney test was 0.0002 (***P < 0.001). (**C–F**) Heme sensing *versus* bacterial establishment in dissected organs. Photograph, and overlap images of photograph and luminescence are shown BALB/c mice infected as in Fig. 2B were euthanized 20 h post-injection. All organs except the brain were imaged in a IVIS 200 system with acquisition times of 10 min and 5 min for WT(pP_gbs0119_-*lux*) and WT(p*lux*) strains respectively; binning 16). No signal was detected in dissected organs of control mice inoculated with the control WT(pP_Ø_-*lux*) strain. In visualization of brain (**F**), no signal was detected using WT(pP_gbs0119_-*lux*), while a strong signal was observed using the bacterial reporter WT(p*lux*). we used acquisition time, 1 min, binning 16; as WT(pP_gbs0119_-*lux*) gave no luminescent signal in the brain (F), only the overlap photo is presented.

The above results (Figs 2 and 4) imply that during infection, GBS concurrently manages host heme toxicity and meets its heme requirements as to ensure respiration metabolism. The WT strain carrying a non-luminescent plasmid (pP_Ø_-*lux*), or the pP_gbs0119_-*lux* plasmid (Table S1) was inoculated intravenously as above. Since our results suggest that GBS uses respiration metabolism during infection (Fig. 4), we verified that P_gbs0119_ was induced by heme in respiration conditions (Fig. S8). Imaging was performed at different times following injection. A significant signal (Fig. 7A) was detected in WT(pP_gbs0119_-*lux*)-infected mice 20 h post-infection. At this late stage of infection, total bacterial luminescence in animals was about 5 times that at 13 h (Fig. S8A) and bacterial counts at this late stage of infection were estimated to be ~6 times those at 13 h (Fig. S8A). The heme sensor-associated signal appeared to be localized in the abdominal region and was significant compared to that in mice injected with the control strain WT(pP_Ø_-*lux*) (Fig. 7A). Statistical analysis confirmed the significance of the luminescent signal in the abdominal region, indicating that GBS responded to heme during infection (Fig. 7B). Surprisingly, a heme sensor signal was absent in the thorax and head regions (Fig. 7A) where GBS preferentially accumulates (Fig. 2B, 13 h or Fig. S8B, 20 h). Nevertheless, examination of dissected organs revealed heme sensor-associated luminescence in the heart (Fig. 7C, left panel). As expected, this localization coincides with that observed with WT(p*lux*) (Fig. 7C, right panel). Weak luminescence driven by P_gbs0119_ compared to the constitutively expressed p*lux* (Fig. 7C), and/or signal obstruction by the rib cage might explain why no luminescence was observed in the heart region in whole animals (Fig. 7A). Similarly, a discrete luminescent signal was present in the cortex of dissected kidneys, correlating with the high bacterial load of this organ (Fig. 7D, right panel). Remarkably, in late stage infection (20 h), heme-sensing-driven luminescence in the liver (Fig. 7E, left panel) exhibited an intensity and distribution similar to that of GBS WT(p*lux*) (Fig. 7E, right panel). No luminescence was detected in the digestive tract, suggesting that abdominal luminescence in living mice arises essentially from the liver. High P_gbs0119_-*lux* expression in the liver contrasts with the relatively low GBS bacterial load (compare luminescence intensity in right panels of Fig. 7C,D,E). These observations seem to suggest that GBS is exposed to toxic levels of heme in this organ. Finally, while the brain was colonized by WT(p*lux*) and generated a strong signal at 20 h post-infection (Fig. 7F, p*lux*), the same strain carrying the heme sensor showed no detectable luminescence (Fig. 7F, pP_gbs0119_-*lux*). This observation correlates with similar CFUs of WT and Δ*hrtBA* strains in the isolated brain (Fig. 3B). We conclude that GBS is exposed to heme toxicity in the heart, kidneys, and liver, but not in the brain.

## Discussion

The dichotomy between toxicity and benefits of heme explains the need for strict management of intracellular heme pools, which may be crucial during infection. In GBS and other bacterial pathogens, homeostasis is mediated by heme efflux *via* HrtBA, whose expression is regulated by HssRS. While HssRS-mediated *hrtBA* regulation is conserved in numerous pathogens, GBS appears to be the first example in which intracellular, rather than extracellular heme is the activating signal^28^. Heme, hemoglobin, and blood all lead to HrtBA induction, suggesting the adaptability of this system to infection conditions. *In vivo* bioluminescent imaging using a set of bacterial and heme sensors identified host compartments in which heme toxicity or utilization impacts GBS survival. Numerous organs, with the exception of the brain, rely on *hrtBA* to limit heme toxicity. Conversely, the use of heme for bacterial respiration impacts infection of all tested organs including the brain. The need for these heme-related functions appears to increase as infection progresses. Bioimaging revealed that GBS colonization of the liver involves strong induction of heme efflux functions late in infection.

Despite conservation of *hrtBA* and *hssRS* genes, their organization and encoded functions in GBS have unique features. First, *hrtBA* and *hssRS* comprise a single operon, whereas in *S. aureus* and other pathogens, *hrtBA* and *hssRS* are organized as 2 independent operons^17^. Only 2 other known Gram-positive bacterial species, (*Granulicatella adjacens* and *Exiguobacterium*, http://string-db.org/) share this organization. The organisation of *hrtBA* and *hssRS* as a single operon implicates that both HssRS expression and activation are controlled by heme. The second unique feature of GBS HrtBA regulation resides in the ECD of HssS. Interestingly, while the 134 amino acids predicted to comprise the ECD of *S. aureus* HssS are implicated in heme signal transduction^28^, in GBS, the ECD is reduced to only 19 amino acid residues, raising doubts on the role of extracellular heme in its activation (Fig. S5). Blast analysis of HssS analogs in several Gram-positive bacteria showed that a reduced ECD is unique to GBS. Our experimental results give strong support that cellular, and not extracellular, heme pools control *hrtBAhssRS* induction and heme efflux (Fig. 6D). We speculate that GBS HssS senses heme through its cytoplasmic and possibly membrane domains by interactions that remain to be investigated. These unique features of *hrtBAhssRS* in GBS support the idea that HrtBA expression control may vary among Gram-positive bacteria as a function of bacterial lifestyle. Our earlier finding that in *L. lactis*, HrtBA is regulated by an intracellular TetR-family heme sensor, HrtR, illustrates this variability^15^. It is thus tempting to speculate that differences in host niches, and in bacterial heme utilization and metabolism, might explain disparities in heme sensing mechanisms that control HrtBA expression. While heme is required for robust GBS virulence and respiration metabolism, the mode of heme entry remains unknown. Despite extensive efforts (mutagenesis, blast analysis, proteomic approach using heme affinity chromatography), we failed to identify heme importers in GBS (our unpublished results). Our studies, including recent work in *L. lactis,* suggest that intracellular heme levels are mainly regulated by heme efflux, and suggest that heme is mainly acquired *via* diffusion. It is tempting to speculate that in conditions of heme excess, e.g., in blood, heme efflux is a main strategy used by GBS to maintain intracellular heme homeostasis.

The infection process likely exposes GBS and other invading pathogens to heme-rich organs. The use of bacterial and heme sensors allowed us to show that in GBS, the HrtBA heme efflux system is required for full virulence and survival in the heart, kidney and liver (Fig. 2). GBS behavior differs markedly from that of *S. aureus,* for which a Δ*hrtA* mutant was more virulent than the WT in a mouse model of systemic infection^17^. *S. aureus* hypervirulence was correlated to an increase of secreted virulence factors in the Δ*hrtA* mutant in response to intracellular heme accumulation^17^. In contrast, attenuated infection by the GBS Δ*hrtBA* mutant implies that it is overcome by toxic amounts of heme in blood and blood-rich cardiac, renal and liver tissues. Free heme concentrations in blood and organs are normally tightly regulated in mammals by a series of mechanisms including dedicated heme-binding proteins that could limit heme availability to invading GBS^11^. Our results therefore suggest that systemic infection by GBS may lead to massive release of free heme, probably due to its hemolytic activity. Direct observation of heme sensing using P_gbs0119_-*lux* showed induction in the same blood-rich organs in which host heme was toxic to GBS establishment. Interestingly, the heme sensor was highly induced in the liver late in infection, suggesting that heme availability is progressively increased in the course of infection. HrtBA-mediated heme efflux may thus be required for GBS survival late in infection. In contrast, deletion of *hrtBA* did not impact the capacity of GBS to colonize cerebral tissue, in keeping with the controlled exchanges between blood and cerebral tissue by the blood brain barrier^30^,^31^. Altogether, these results underline the crucial role of host heme homeostasis control for GBS adaptation *in vivo*.

GBS does not biosynthesize heme, but it relies on exogenous heme to activate a respiration metabolism *in vitro*. In contrast to pathogens such as *S. aureus* that depend on host heme as an iron source, GBS has low iron requirements and does not encode identified heme-oxygenases^13^. These observations raise the question of whether environmental heme constitutes a metabolic requirement for GBS during infection. A role for bacterial respiration in virulence was reported for diverse Gram-positive pathogens, including *S. aureus*^32^ and *Mycobacterium tuberculosis*^33^, but unlike GBS, these bacteria biosynthesize heme and are autonomous for respiration. Our present and previous results give strong evidence that exogenous heme-activated respiration metabolism is required for successful infection^20^. This result correlates here with impaired colonization by the GBS Δ*cydA*(p*lux*) strain of organs usually targeted by GBS, i.e., the heart, kidney and brain. Thus, while *in vitro* GBS heme requirements are facultative, they are essential and obligatory *in vivo*. We hypothesize that respiration metabolism increases bacterial robustness and reduces heme toxicity (by its incorporation in cytochrome oxidase), both of which might improve GBS fitness and survival in the hostile host environment. Identification of heme as a key player of GBS virulence could lead to novel antimicrobial strategies that inhibit GBS respiration or HrtBA-related functions.

## Methods

### Ethics statement

Animal experiments were carried out in strict accordance with the recommendations in the guidelines of the Code for Methods and Welfare Considerations in Behavioural Research with Animals of the EEC council (Directive 2010/63/EU). The protocols were approved by the Animal Care and Use Committee at the Research center of Jouy en Josas (COMETHEA; protocol number 15–61) and by the Ministry of Education and Research (APAFIS#2277-2015081917023093 v4). All efforts were undertaken to minimize animal suffering. All experimental procedures were performed in biosafety level 2 facilities.

### Bacterial strains

GBS strain NEM316, whose genome sequence is known, belongs to the capsular serotype III strain (GBS) and was isolated from a case of fatal septicemia^34^. The GBS Δ*hrtBA* and Δ*hssRS* mutants were constructed as described in S1 Text and S1 Table. The Δ*cydA* mutant was generated as described previously^35^. Characteristics of relevant plasmids are described in S1 Table.

### Bacterial Growth Conditions and Media

GBS and its derivatives were grown as overnight (ON) precultures at 37 °C in rich M17 liquid broth (DIFCO) supplemented with 0.2% glucose. Heme content of this medium is below 0.5 μM (data not shown), and is insufficient to activate heme-related functions in GBS. Precultures were diluted in M17 medium supplemented with 1% glucose for further use in fermentation conditions. When indicated, GBS WT and mutants were plated on M17/agar plates supplemented with 1% glucose or blood plates (Columbia agar, 5% sheep blood; Biomerieux, France). *E. coli* strains were grown in Luria-Bertani (LB) medium at 37 °C with aeration by shaking at 180 rpm. When needed, antibiotics were used as follows: 50 μg/ml kanamycin and 10 μg/ml chloramphenicol for *E. coli*; 5 μg/ml erythromycin and 5 μg/ml chloramphenicol for GBS. Hemin was prepared from a stock solution of 10 mM hemin chloride dissolved in 50 mM NaOH; Frontier Scientific, USA). 10 mM Hb (Sigma, Saint-Louis) stock solution was prepared freshly in PBS. Fresh heparinized mouse blood (BALB/c) was purchased from Janvier laboratory (France) and used within 24 h of blood withdrawal.

### β-galactosidase assays

β-galactosidase (β-gal) activity was assayed on bacteria grown as described in the previous section. Briefly, GBS strains were grown to OD_600nm_ = 0.5 and then incubated for 1 h with the indicated concentrations of hemin or Hb and for 3 h in the case of blood. β-galactosidase activity was quantified by luminescence in a infinite M200 luminescence reader (TECAN, Germany) using the β-glo assay system as described^15^. All β-galactosidase results represent the mean ± standard deviation from triplicate samples and are representative of 3 independent experiments

### Heme toxicity assay on plates

Stationary phase cultures were diluted 1/10 with 0.6% melted agar in H_2_O and plated above solid M17 medium containing 1% glucose. 10 μl of hemin (10 mM) was pipetted directly onto the mixture bacteria and agar, and incubated ON at 37 °C.

### Cellular status of heme

Bacteria were grown to OD_600nm_ = 0.5 prior to addition or not of 2 μM ^57^FePPIX (Frontier Scientific, USA) for 1 h. Cells were washed 3 times in PBS. Cell pellets were dessicated and mineralized by successive incubations in 65% nitric acid solution. ^57^Fe was quantified by inductively coupled plasma mass spectroscopy (Agilent 7700X, USA).

### Mouse virulence assay

For systemic injection, GBS strains were prepared as follows: GBS precultures were diluted and grown in M17 with 1% glucose to OD_600nm_ = 0.5 that was determined to correspond to 6.10^8^ CFU/ml. Bacteria were then centrifuged at 6000 rpm at 4 °C for 15 min and pellets were resuspended in PBS to a final concentration of 2.10^8^ cells/ml. Bacterial stocks were aliquoted and frozen in liquid nitrogen. Aliquots were kept at −80 °C until use. Bacterial counts were confirmed by plating serial dilutions of cultures. 6 week old female BALB/c mice (Janvier, France) were anesthetized intraperitonally with 100 μg/g ketamine and 15 μg/g xylazine. For systemic inoculation, 2.10^7^ bacteria in a 100 μl volume were injected into the retro-orbital vein of the right eye. When indicated the ventral area of animals was shaven prior to infection. Detection of luminescence (see below) in whole animals (0 time point) was performed immediately following injection (see above). Detection of light emission at time 0 (strains carrying the p*lux* plasmid) indicated that were not successfully injected (luminescence localized mostly in the head around the injection site); such individuals were excluded from the experiment and immediately euthanized. Following image acquisition, mice were removed from the IVIS 200 imaging system and immediately sacrificed by cervical dislocation. When indicated, the animals were dissected for imaging of the isolated organs or determination of CFU. For that purpose, each organ was homogenized with an Ultra-Turrax (IKA Works, Germany) in 2 ml PBS and bacterial load within each organ was quantified by plating serial dilutions of the organ homogenate and counting CFU. Prism 5 (GraphPad Software, La Jolla, CA) was used for statistical analyses as indicated in the figure legends.

### *In vivo* imaging

Light emission from whole animals was measured in an *in vivo* imaging system (IVIS 200, Caliper Life Sciences, USA) equipped with the Living image software (version 4.0, Caliper Life Science, USA). IVIS 200 was also used to evaluate luminescence on agar plates or from isolated organs (see above). Bioluminescence images were acquired with a 25 cm field of view (FOV), medium or large binning factor and an exposure time as indicated. A digital false-color photon emission image was generated according to photon counts within a constant region of interest (ROI) corresponding to the surface of the entire mouse. Rainbow images show the relative level of luminescence ranging from low (blue), to medium (green), to high (yellow/red). Photon emission was measured in radiance (p.s^−1^.cm^−2^ sr^−1^). Threshold parameters were chosen to maintain the luminescence detection under saturation level and were kept identical within an experiment. Images were adjusted for brightness and contrast using PhotoShop CS3 (Adobe Systems, San Jose, CA) with parameters kept identical in all images of the same figure. Quantitative analysis of bioluminescence of whole bodies or selected abdominal areas (in the case of the heme sensor) corresponding to a selected ROI was performed taking into account the contribution of the background light emission. ROI measurements are expressed in total flux of photons (p/s).

## Additional Information

**How to cite this article**: Joubert, L. *et al*. Visualization of the role of host heme on the virulence of the heme auxotroph *Streptococcus agalactiae. Sci. Rep.* **7**, 40435; doi: 10.1038/srep40435 (2017).

**Publisher's note:** Springer Nature remains neutral with regard to jurisdictional claims in published maps and institutional affiliations.

## Supplementary Material

supplementary Data and Figures
