# Supplementary material for: Visualization of the role of host heme on the virulence of the heme auxotroph Streptococcus agalactiae
Source: Sci Rep. 2017 Jan 16;7:40435. doi: 10.1038/srep40435 (PMC5238366; doi:10.1038/srep40435)
Supplement: supplementary Data and Figures [file srep40435-s1.pdf]

Visualization of the role of host heme on the virulence of the heme auxotroph *Streptococcus agalactiae*

Laetitia Joubert, Jean-Baptiste Dagieu, Annabelle Fernandez, Aurélie Derré-Bobillot, Elise Borezée-Durant, Isabelle Fleurot, Alexandra Gruss and Delphine Lechardeur

Supplementary informations

Supplementary Methods

Plasmid constructions. Plasmid $pP_{Ø}$-*lux* (pTCV-J22, Table S1) was constructed using the Gibson assembly method[1]. Briefly, the *luxABCDE* operon was PCR-amplified using pXen5 as a template with oligonucleotides O1 and O2 (Table S2). The entire backbone except the *lac* gene of the pTCV-*lac* plasmid was PCR-amplified with O3 and O4 (Table S2). The 2 resulting DNA fragments were ligated as described[1].

Plasmids $pP_{gbs0119}$-*lux* (pTCV-J23) and p*lux* (pTCV-J24) were constructed as follows: DNA fragments containing $P_{gbs0119}$ were PCR amplified from $pP_{gbs0119}$-*lac* (pTCV-J21) and P_{23} from p*hrtBA*[2] with the primer pairs O5/O6 and O7/O8 respectively (Tables S1 and S2). The 2 PCR products were gel purified, digested with *Eco*RI and *Bam*H1 and ligated with the plasmid $pP_{Ø}$-*lux* (pTCV-J22, Table S1).

The pP_{23}-*hrtBA*, $P_{gbs0119}$-*lac* (pTCV-J25, Table S1) was obtained by an overlap of 3 PCRs : P_{23} amplified from p*hrtBA*[2] with primer O7/O9; *hrtBA* and $P_{gbs0119}$ from genomic DNA of GBS NEM316 with primers O10/O11 and O12/O6. The resulting PCR product was digested by *Eco*R1 and *Bam*H1 and ligated into the *Eco*R1 and *Bam*H1 restriction sites of pTCV-*lac* (Tables S1 and S2).

The plasmid $pP_{gbs0119}$-*hssRS-HA* (pTCV-J28) was obtained by an overlap of 2 PCRs following by an extension of the 5' extremity of the PCR product to include the sequence of the HA hemagglutinin influenza peptide (HA) sequence corresponding to the YPYDVPDYA epitope. The $P_{gbs0119}$ promoter and *hssRS* genes were PCR amplified from GBS NEM316 genomic DNA with primers O21/O22 and O23/O24 respectively. The resulting DNA fragments were combined by PCR overlap with the oligonucleotides O21/O24 to obtain the $P_{gbs0119}$-*hssRS* DNA fragment. The HA sequence was added by PCR with O21/O25. The resulting PCR

product was digested by *Eco*R1 and *Bam*H1 and ligated into the *Eco*R1 and *Bam*H1 restriction sites of pTCV-*lac*.

The plasmid pP_{23}-*hrtBA*, P_{hrt}*hrtR-lac* (pTCV-J30, Table S1) was obtained by an overlap of 2 PCRs: the first PCR amplified P_{23} from p*hrtBA*[2] with primer O7 (*Eco*RI) and O9 (Tables S1, S2). The second PCR amplified *hrtBA* from GBS NEM316 genomic DNA with primer O10 and O26 (*Eco*R1). Overlap PCR between the two PCR fragments was obtained using oligonucleotides O7 and O26 (Table S2). The PCR product was digested by *Eco*R1 and inserted into the plasmid pP_{hrt}*hrtR-lac* (pTCV-J29) (Table S1).

All inserts were sequenced.

Construction of GBS Δ*hrtBA* and Δ*hssRS* mutant strains. The Δ*hrtBA* GBS (NEMJ18, Table S1) mutant strain was obtained by PCR amplification of 2 fragments of ~ 450 bp flanking the *gbs0119-0120* genes using primer pairs O13/O14 and O15/O16 respectively (Table S2). Following digestion by *Bam*HI, both fragments were ligated together and purified, and the resulting ligation product was amplified using O13 and O16. The amplicon was then digested by *Eco*RI and cloned into the temperature-sensitive shuttle vector, pG+host1[3] giving rise to the pΔ*hrtBA* plasmid (pTCV-J26, Table S1). The GBS Δ*hssRS* mutant strain (NEMJ19, Table S1) was constructed as follows: 2 fragments of ~ 500 bp flanking the *gbs0121-0122* genes were amplified from genomic DNA of GBS NEM316 by PCR using primer pairs O17/O18 and O19/O20 (Table S2). The 2 DNA fragments were fused together by PCR-overlap with primers O17 and O20. The resulting 1.1 kb fragment was gel-purified, digested by *Sal*I and *Eco*RI, and cloned into pG+host1[3] giving rise to the pΔ*hssRS* plasmid (pTCV-J27, Table S1). The recombinant vectors were introduced by electroporation into GBS strain NEM316 at 28°C. The double-crossover events leading to the expected gene disruptions were obtained as described[3]. Correct inactivation was confirmed by PCR analysis with oligonucleotides hybridizing outside the region of the double-crossover events. The mutants pTCV-J26 and pTCV-J27 are respectively referred to as Δ*hrtBA* and Δ*hssRS* in the manuscript (Table S1).

Immunoblots. Immunoblotting was performed on bacterial lysates of strains transformed with the indicated expression vectors as reported previously[4] using the mouse monoclonal anti-HA.11 (Covance, New Jersey).

Respiration metabolism. Induction of respiration metabolism in GBS was obtained by supplementing M17 growth medium with 1 µM hemin and 10 µM K2 vitamin as reported earlier[4].

References

1 Gibson, D. G. *et al.* Enzymatic assembly of DNA molecules up to several hundred kilobases. *Nature methods* **6**, 343-345, doi:10.1038/nmeth.1318 (2009).

2 Lechardeur, D. *et al.* Discovery of an intracellular heme-binding protein, HrtR, that controls heme-efflux by the conserved HrtB HrtA transporter in *Lactococcus lactis*. *J. Biol. Chem.* **287**, 4752-4758, doi:10.1074/jbc.M111.297531 (2012).

3 Biswas, I., Gruss, A., Ehrlich, S. D. & Maguin, E. High-efficiency gene inactivation and replacement system for gram-positive bacteria. *Journal of bacteriology* **175**, 3628-3635 (1993).

4 Yamamoto, Y. *et al.* Respiration metabolism of Group B Streptococcus is activated by environmental haem and quinone and contributes to virulence. *Mol Microbiol* **56**, 525-534, doi:10.1111/j.1365-2958.2005.04555.x (2005).

Supplementary Tables

Table S1. Strains and plasmids used in this study.

Strain/plasmid	***Characteristics***	***Source/ reference***
Strain		
E. coli		
TOP10	*F⁻ mcrA Δ(mrr-hsdRMS-mcrBC) ∅80lacZΔM15 lacX74 recA1 deoR araD139 Δ(ara-leu)7697 galU galK rpsL (Str^r) endA1*	Invitrogen
S. agalactiae		
NEM316	Serotype III isolated from neonatal blood culture	[1]
NEMJ17	NEM316 *cydA::aph*A3, kan^R	[2]
NEMJ18	NEM316 Δ*hrtBA*, deletion of *hrtB* and *hrtA* genes	This study
NEMJ19	NEM316 Δ*hssRS*, deletion of *hssR* and *hssS* genes	This study
Plasmid		
pTCV-*lac*	Conjugative *E. coli* Gram-positive bacteria shuttle plasmid with β-galactosidase reporter construct. Ery^R Kan^R	[3]
pXen5	Conjugative *E. coli* Gram-positive bacteria shuttle plasmid with *luxABCDE* reporter construct. Ery^R	Perkin Elmer
pTCV-J21	p$P_{gbs0119}$-*lac*, gbs0119 promoter region cloned into pTCV-*lac*. Ery^R Kan^R	[4]
pTCV-J22	p$P_{Ø}$-*lux*, *luxABCDE* reporter genes Ery^R Kan^R	This study
pTCV-J23	p$P_{gbs0119}$-*lux*, *gbs0119* promoter cloned in pTCV-J22. Ery^R Kan^R	This study
pTCV-J24	p*lux*, P_{23} promoter cloned in pTCV-J23. Ery^R Kan^R	This study
pTCV-J25	pP_{23}*hrtBA*,$P_{gbs0119}$-*lac*,expression of *hrtBA* with the constitutive promoter $P_{23,}$ cloned into pTCV-J21	This study
pTCV-J26	pΔ*hrtBA, hrtBA* fragment cloned into pG+host1 to obtain the Δ*hrtBA* mutant	This study
pTCV-J27	pΔ*hssRS, hssRS* fragment cloned into pG+host1 to obtain the Δ*hssRS* mutant	This study
pTCV-J28	p$P_{gbs0119}$-*hssRS-HA*, expression of *hssRS* with the $P_{gbs0119}$ promoter cloned into pTCV-*lac*. HssS is tagged at its Ct with the HA epitope. Ery^R Kan^R	This study
pTCV-J29	pP_{hrt}*hrtR-lac,* DNA fragment containing the promoter region of *Lactococcus lactis hrtRBA* operon and *hrtR* cloned in pTCV-*lac*. Ery^{R}Kan^R	5
pTCV-J30	pP_{23}*hrtBA*,P_{hrt}-hrtR-*lac*,expression of *hrtBA* with the constitutive promoter $P_{23,}$ cloned into pTCV-*lac*	This study

Table S2. Oligonucleotides used in this study.

Primer	*Sequence 5'-3'*	*Target*
**O1**	CTTTTATATAGGGAAAAGGTGGTGAGAAGCTTCTGCAG ATGAAGCAAGAG	*luxABCDE*
**O2**	CTTAACCGAAGCGTTTGATAGTTAATCTTTATTTATCAG CCG GAAAACCT	*luxABCDE*
**O3**	AGGTTTTCCGGCTGATAAATAAAGATTAACTATCAAAC GCTTCGGTTAAG	pTCV-*lac*
**O4**	CTCTTGCTTCATCTGCAGAAGCTTCTCACCACCTTTTCC CTATATAAAAG	pTCV-*lac*
**O5**	ATACGCCAGAATTCTCGGCGAC	$P_{gbs0119}$
**O6**	CCTTGGATCCTTTGATGTGAAC	$P_{gbs0119}$
**O7**	CCGGAATTC CGTTCCTAAAAAGGAATAAGC	P_{23}
**O8**	CGCGGATCCCTAGAATTTAATTCTAATACT	P_{23}
**O9**	CATGTGACATGGTTTTTCTCCTTTGTAGAATTTAATTCT AATACTATTTT	P_{23}
**O10**	AAAATAGTATTAGAATTAAATTCTACAAAGGAGAAAAA CCATGTCACATG	*hrtBA*
**O11**	AACAAGAGCCATTTAGAAGAAGAGTTTAGAAGT TTTCTTTAACTAATTTC	*hrtBA*
**O12**	GAAATTAGTTAAAGAAAACTTCTAAACTCTTCTTCTAAA TGGCTCTTGTT	$P_{gbs0119}$
**O13**	TAGGAATTCTTCAACTCCTC	*gbs0119*
**O14**	ATTGGATTCAAGCATGTGACA	*gbs0119*
**O15**	TGGGGATTCACGATGAGCGC	*gbs0120*
**O16**	AGTTTTTATCAGTTTTCCATTTTA	*gbs0120*
**O17**	CCGGTCGACTATGTCAATCATGAGAACGTT	*gbs0121*
**O18**	TTGGCAGTTATTGTTTTAAATAATAAATCACCTCTAGCT TATTG	*gbs0121*
**O19**	CAATAAGCTAGAGGTGATTTATTATTTAAAACAATAAC TGCCA	*gbs0122*
**O20**	CGGGAATTCCACTTGGTCATTTCCTTTAC	*gbs0122*
**O21**	TTAAATGGATCCCTTGTCTAATCGTACTCA	$P_{gbs0119}$
**O22**	TCAAAAGATCAAAGGAGAAAAACCATGATTAAAATATT AGTTGTAGAGGA	$P_{gbs0119}$
**O23**	TCCTCTACAACTAATATTTTAATCATGGTTTTTCTCCTTT GATCTTTTGA	*hssR*
**O24**	AATCTGGAACATCATATGGATACATTGATAAAGGTAGT TCTACACAAAAT	*hssS-HA*
**O25**	AATTAAGAATTCTTAAGCATAATCTGGAACATCAT ATGGATACAT	*HA*
**O26**	CAAATAGATATGAACAATGAATTCCG	*hrtBA*

References

1. Gasson, M. J. Plasmid complements of *Streptococcus lactis* NCDO 712 and other lactic streptococci after protoplast-induced curing. *Journal of bacteriology* **154**, 1-9 (1983).
2. Yamamoto, Y. *et al.* Roles of environmental heme, and menaquinone, in Streptococcus agalactiae. *Biometals : an international journal on the role of metal ions in biology, biochemistry, and medicine* **19**, 205-210, doi:10.1007/s10534-005-5419-6 (2006).
3. Maguin, E., Prevost, H., Ehrlich, S. D. & Gruss, A. Efficient insertional mutagenesis in lactococci and other gram-positive bacteria. *Journal of bacteriology* **178**, 931-935 (1996).
4. Fernandez, A. *et al.* Two coregulated efflux transporters modulate intracellular heme and protoporphyrin IX availability in *Streptococcus agalactiae*. *PLoS Pathog.* **6**, e1000860, doi:10.1371/journal.ppat.1000860 (2010).

5 Lechardeur, D. *et al.* Discovery of an intracellular heme-binding protein, HrtR, that controls heme-efflux by the conserved HrtB HrtA transporter in *Lactococcus lactis*. *J. Biol. Chem.* **287**, 4752-4758, doi:10.1074/jbc.M111.297531 (2012).

Supplementary Figure legends

Figure S1. Hemin toxicity. (**A-C**) Growth of WT (A), Δ*hrtBA* (B) and Δ*hrtBA*(P23-*hrtBA*) (C) in the presence of hemin. Precultures of the indicated bacteria grown in M17 with 0.2 % glucose were diluted to OD_{600nm} = 0.01 in a 96 well plate (200 µl M17 with 1 % glucose with the indicated concentration of hemin). OD_{600nm} was measured at 37°C each 30 min during 12 h in an infinite M200 spectrolumineter (TECAN, Austria). Results are representative of three independent experiments.

Figure S2. Evaluation of luminescent GBS strains for *in vivo* experiments. (**A**) p*lux* stability in GBS. WT(p*lux*) was grown ON at 37°C in M17 supplemented with 0.2 % glucose and 3 µg/ml erythromycin. The culture was then diluted 1/1000 in M17 supplemented with 1 % glucose without antibiotics and grown over a 5 days period with a serial dilution every 24h. Aliquots were taken every day for CFU determinations on solid medium containing or not 3 µg/ml erythromycin. Plasmid stability was calculated as the percentage of antibiotic resistant cells bacteria compared to the total CFU. (**B**) Correlation between CFU and luminescence. A GBS WT(p*lux*) preculture was diluted to an OD_{600nm} = 0.01 in M17 supplemented with 1 % glucose and grown at 37°C. At different times ranging from 0 to 8 h, 200 µl of the culture was removed, luminescence was quantified in a Biolumate LB9500T luminometer (Berthold, Germany) and CFU were determined by serial dilution and plating on agar plates containing 3 µg/ml erythromycin.(**C**) Growth profile of WT and WT(p*lux*) GBS strains. Precultures of the indicated bacteria grown in M17 with 0.2 % glucose were diluted to an OD_{600nm} = 0.01 and grown as in Fig. S1A. Results are representative of 3 independent experiments.

Figure S3. ***In vitro* evaluation of bioluminescence of WT(p*lux*), Δ*hrtBA*(p*lux*) and Δ*cydA*(p*lux*).** (**A**) Evaluation of luminescence emission by WT(p*lux*) and Δ*hrtBA*(p*lux*) during growth in liquid culture. Precultures of the indicated bacteria grown in M17 with 0.2 % glucose were diluted to an OD_{600nm} = 0.01 in 200 µl M17 with 1 % glucose in a 96-well white plate with clear bottom (Greiner). OD_{600nm} and luminescence were measured each 30 min during 10 h in an infinite M200 spectrolumineter (TECAN, Austria). Results are representative of 3 independent experiments. (**B**) Evaluation of luminescence emission by WT (p*lux*) and Δ*cydA*(p*lux*) during growth. Luminescence was evaluated as in (B).

Figure S4. Heme-dependent induction of the $P_{gbs0119}$ promoter. (**A-C**) Growth of WT (A), Δ*hssRS* (B) and Δ*hssRS* (p$P_{gbs0119}$-*hssRS-HA*) (C) in the presence of hemin. Precultures of the indicated bacteria grown in M17 with 0.2 % glucose were diluted to OD_{600nm} = 0.01 in a 96 well plate (200 µl M17 with 1 % glucose with the indicated concentration of hemin). OD_{600nm} was measured at 37°C each 30 min during 12 h in an infinite M200 spectrolumineter (TECAN, Austria). Results are representative of three independent experiments. (**D**) WT and Δ*hrtBA* GBS strains transformed with $P_{gbs0119}$-*lac* were grown to OD_{600nm} = 0.5. β-galactosidase activity was induced for 1 h in the presence of the indicated concentrations of hemin and quantified by luminescence (Materials and Methods). Results represent the mean ± standard deviation from triplicate samples. (**E**) Evaluation of luminescence emission by WT ($P_{gbs0119}$-*lux*) during growth in liquid culture. Precultures of the indicated bacteria grown in M17 with 0.2 % glucose were diluted to an OD_{600nm} = 0.01 in 200 µl M17 with 1 % glucose in a 96-well white plate with clear bottom with the indicated concentration of hemin. OD_{600nm} and luminescence were measured each 30 min during 10 h in an infinite M200 spectrolumineter (TECAN, Austria). Results are representative of 3 independent experiments.

Figure S5. Comparison of *S. aureus* Newman and GBS NEM316 HssS amino acid sequences and membrane topologies. Alignments were determined with the EMBL-EBI multiple sequence alignment program MUSCLE (http://www.ebi.ac.uk/Tools/msa/muscle/). The transmembrane topology prediction of the proteins was established with TopPred 1.10 program through the Pasteur Institute MOBYLE portal (http://mobyle.pasteur.fr). Cytoplasmic domains are indicated in black, transmembrane helixes in orange, and the extracellular loop (ECD) in green.

Figure S6. Activation of the *Lactococcus lactis* intracellular heme sensor reporter (P_{hrt}*hrtR-lac*) in GBS. WT, Δ*hrtBA* and Δ*hrtBA*(P_{23}-*hrtBA*) carrying the intracellular heme sensor reporter plasmid (pP_{hrt}*hrtR*-lac) were grown to OD_{600nm} = 0.5 and incubated with 1 µM hemin for 1 h at 37°C. Bgal expression was determined as described above. β-galactosidase activity was quantified by luminescence (Methods). Results represent the mean ± standard deviation from triplicate samples and are representative of 3 independent experiments.

Figure S7. **Induction of Pgbs0119 in GBS WT and Δ*cydA* GBS strain.** WT (NEM316) and Δ*cydA* (NEMJ19) GBS strains transformed with $P_{gbs0119}$-*lac* were grown in or respiration growth conditions (1 µM hemin, 10 µM K2 vitamin, aeration) to OD_{600nm} = 0.5. β-

galactosidase activity was induced for 1 h in the presence of 1 µM hemin and quantified by luminescence (Methods).

Figure S8. Quantification of bioluminescence in live mice. (**A**) GBS WT(p*lux*) luminescence in mice 20 h following the inoculation of GBS WT(p*lux*). BALB/c mice were infected with 2.10^7 CFU of WT(p*lux*) as described in Fig. 3 and Fig. 4. The luminescence was determined with the IVIS 200 imaging system (acquisition time, 5 min; binning 16). Images were analyzed by measuring the total light flux (number of photons per second) as in Fig. 2C. To show progression of infection between 13 h and 20 h, data from Fig. 3B were reported on the same graph. Values correspond to batches of 25 mice for each time point. Two tailed Mann-Whitney was used to determine P values: 8 h/13 h, P = 0.0001; 13 h/20 h, P = 0.0075 (**, $P < 0.01$; ***, $P < 0.001$). (**B**) Overall luminescence of anesthetized mice infected with GBS WT(p*lux*) at 20 h post-infection (as in B). To visualize relative bacterial loads in the head/thorax regions compared to the abdomen, the bioluminescence sensitivity threshold was lowered on acquired images (acquisition time, 5 min; binning 16). Results are representative of 3 experiments.

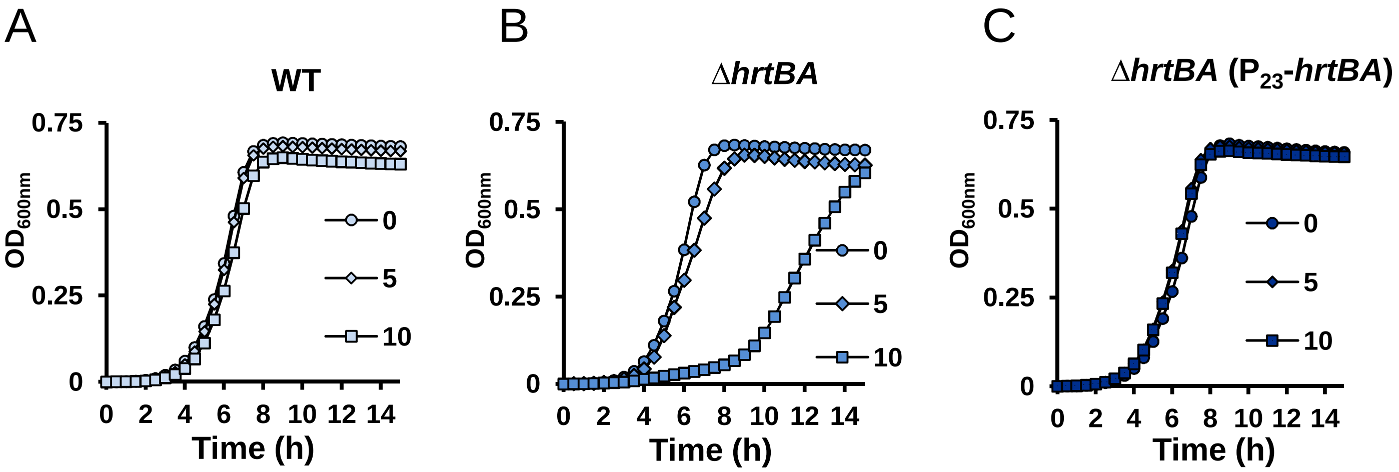

A
WT
0.75
0.5
0.25
0
OD600nm
0
5
10
0 2 4 6 8 10 12 14
Time (h)
B
ΔhrtBA
0.75
0.5
0.25
0
OD600nm
0
5
10
0 2 4 6 8 10 12 14
Time (h)
C
ΔhrtBA (P23-hrtBA)
0.75
0.5
0.25
0
OD600nm
0
5
10
0 2 4 6 8 10 12 14
Time (h)

FIGURE S1

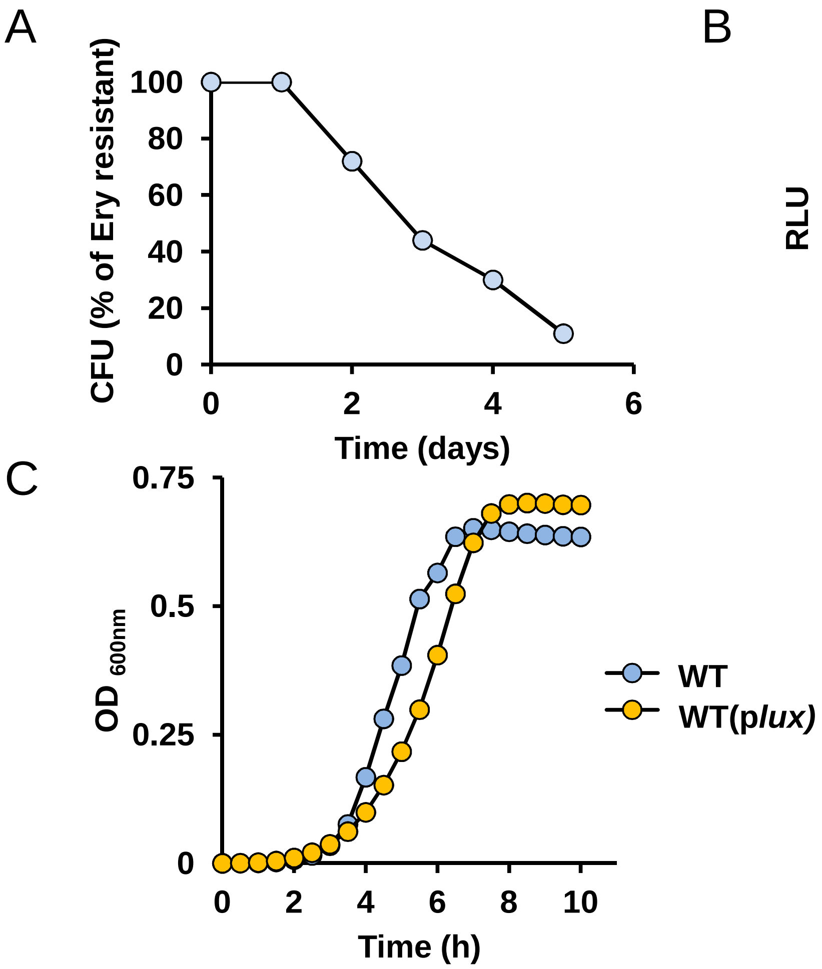
A
CFU (% of Ery resistant)
100
80
60
40
20
0
0
2
4
6
Time (days)
B
15
10
5
0
RLU
0
0.5
1
1.5
CFU
C
0.75
0.5
0.25
0
OD 600nm
0
2
4
6
8
10
Time (h)
WT
WT(plux)

FIGURE S2

A

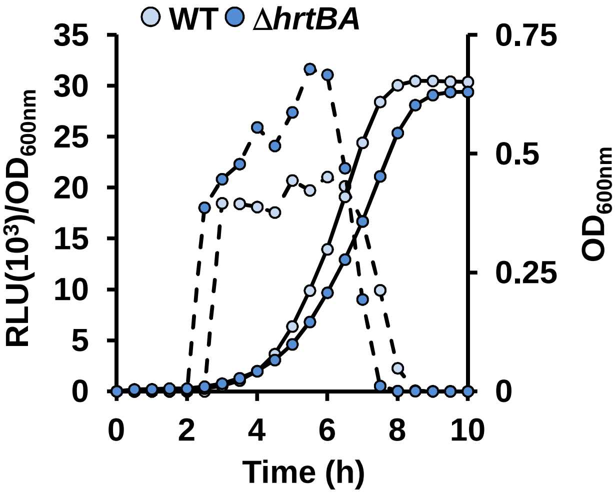
WT ΔhrtBA
RLU(10³)/OD600nm
OD600nm
Time (h)
35
30
25
20
15
10
5
0
0.75
0.5
0.25
0
0 2 4 6 8 10

B

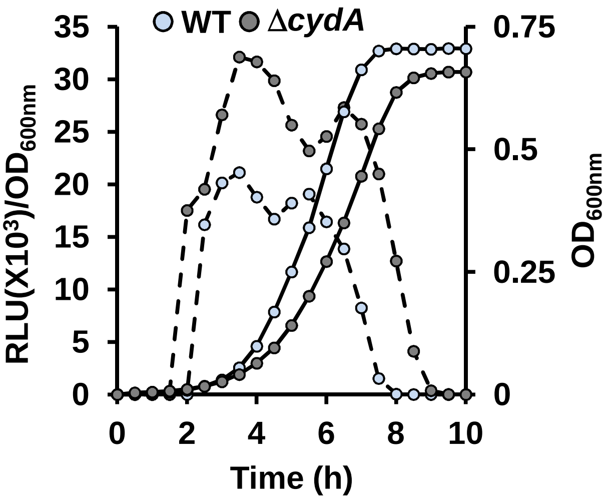
WT ΔcydA
RLU(X10³)/OD600nm
OD600nm
Time (h)
35
30
25
20
15
10
5
0
0.75
0.5
0.25
0
0 2 4 6 8 10

FIGURE S3

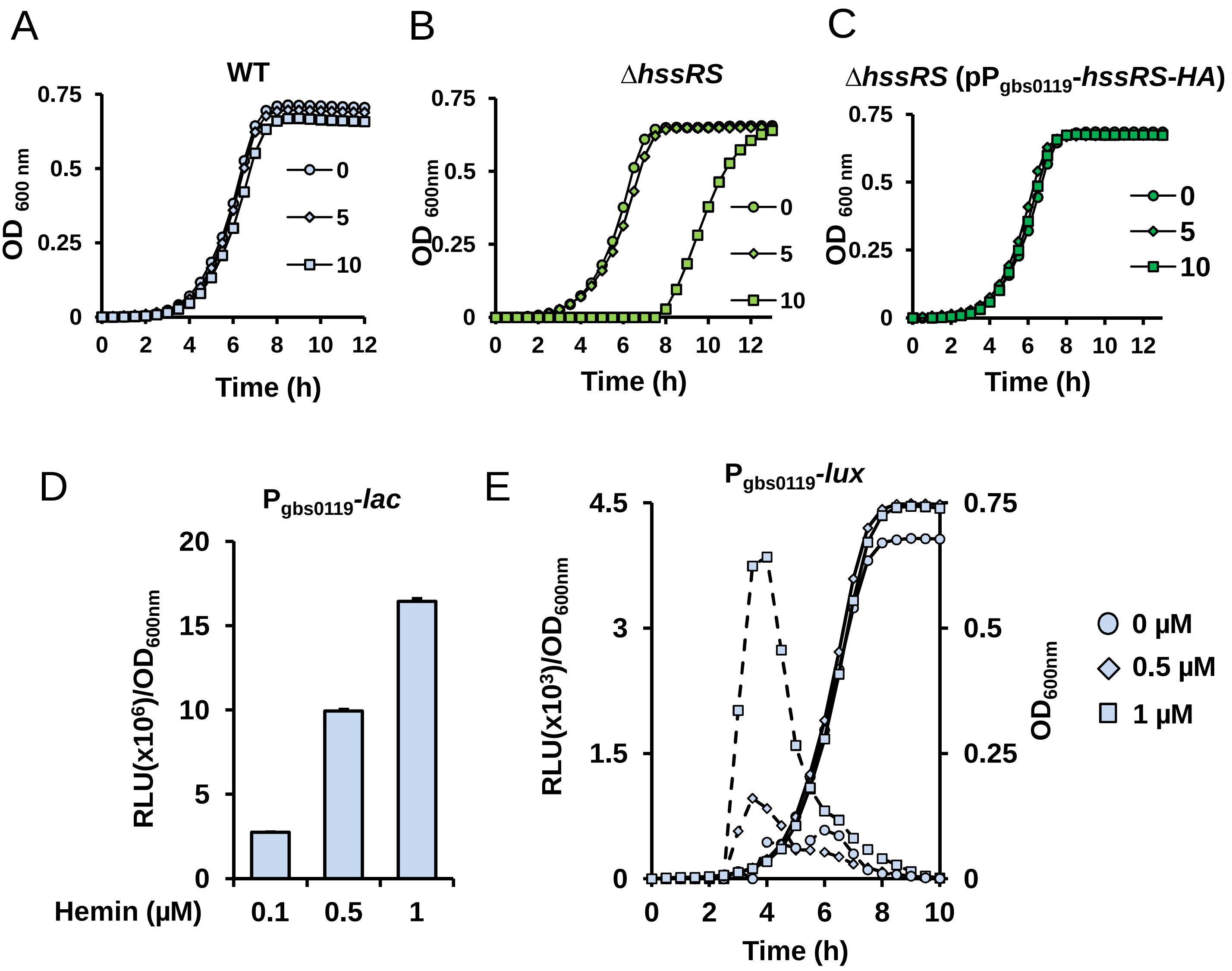
A
WT
0.75
0.5
0.25
0
OD 600 nm
0
5
10
0 2 4 6 8 10 12
Time (h)
B
ΔhssRS
0.75
0.5
0.25
0
OD 600nm
0
5
10
0 2 4 6 8 10 12
Time (h)
C
ΔhssRS (pPgbs0119-hssRS-HA)
0.75
0.5
0.25
0
OD 600 nm
0
5
10
0 2 4 6 8 10 12
Time (h)
D
Pgbs0119-lac
20
15
10
5
0
RLU(x106)/OD600nm
Hemin (µM)
0.1 0.5 1
E
Pgbs0119-lux
4.5
3
1.5
0
RLU(x103)/OD600nm
0.75
0.5
0.25
0
OD600nm
0 µM
0.5 µM
1 µM
0 2 4 6 8 10
Time (h)

FIGURE S4

```
NWMN_2264       MFKTLYARIAIYSITVILFSALISFVLTNVYYHYNLKASNDAKIMKTLKE 50
GBS0122         MTKLRRFRFPLR------FYFTLMFVLT-------MLFSVLASLLLVAAI 37
                * *    *:.:       *   : ****       :  *  *.:: .

NWMN_2264       ARQYEQSAKPTHIQQYFKHLGQMNYQIMTIDQKGHKTFYGEPFREDTLSQ 100
GBS0122         VFTFFQGVLTTHVLQVS--------------------------------- 54
                .  : *.. .**: *

NWMN_2264       NAINNVLNNQDYHGIKDKPFALFVTGFFDNVTDNTVGINFKTKDGSIAVF 150
GBS0122         --------------------------------------------------

NWMN_2264       MRPDIGETFSEFRTFLAVLLMLLLFISISLVIASTYSIIRPVKKLKLATE 200
GBS0122         --------------ALAVVFLSLVIASISMWYG-SYHLTKPILDISRIVS 89
                               ***::: *:: ***:  . :* : :*: .:.  ..

NWMN_2264       RLIDGDFETPIKQTRK--------DEIGTLQYHFNKMRESLGQVDQMRQH 242
GBS0122         NVADGDFEGHIYRNSNRRKSYEYYNELDELSESINQMIVSLSHMDHMRKD 139
                .: *****  * :. :        :*:. *.  :*:*  **.::*:**:.

NWMN_2264       FVQNVSHEIKTPLTHIHHLLSELQQT-SDKTLRQQYINDIYTITTQLSGL 291
GBS0122         FITNVSHELKTPIAAVANIVELLQDPELDEETQSELLGLVKTESLRLTRL 189
                *: *****:***:: : :::. **:.  *:  :.: :. : * : :*: *

NWMN_2264       TTELLLLSELDNHQHLLFDDKIQVNQLIKDIIRHEQFAADEKSLIILADL 341
GBS0122         CDTMLQMSRVDNQETIGELSSVRVDEQIRQAMISLTERWQAKRINFQLDS 239
                   :* :*.:**:: :   ..::*:: *:: :       : * : :  *

NWMN_2264       ESINFLGNQRLLHQALSNLLINAIKYTDVGGAIDIALQHSHNNIIFTISN 391
GBS0122         KPYTVYSNSDLLMQVWINLLDNAIKYSDDIVDLRVRMEETNNHYLRVIIS 289
                :. .. .*. ** *.  *** *****:*    : : ::.::*: : .* .

NWMN_2264       D-GSPISPQAEARLFERFYKVSKHDN--SNGLGLAITKSIIELHHGTIQF 438
GBS0122         DKGRGISQYDVQHIFDKFYQADQSHNQQGNGLGLAIVKRIIVLCKGRISV 339
                * *  **     ::*::**:..: .*  .*******.* ** * :* *..

NWMN_2264       TQSNEYVTTFTITLPNNSL 457
GBS0122         SSQFEIGTEFCVELPLS-- 356
```

FIGURE S5

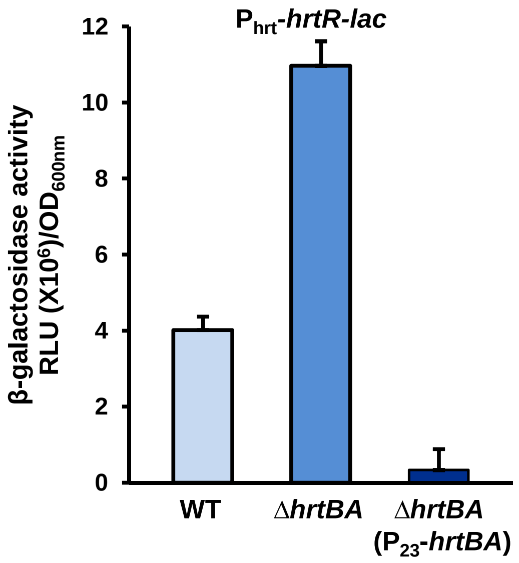

P$_{hrt}$-*hrtR-lac*
β-galactosidase activity
RLU (X10^{6})/OD$_{600nm}$
12
10
8
6
4
2
0
WT
Δ*hrtBA*
Δ*hrtBA*
(P$_{23}$-*hrtBA*)

FIGURE S6

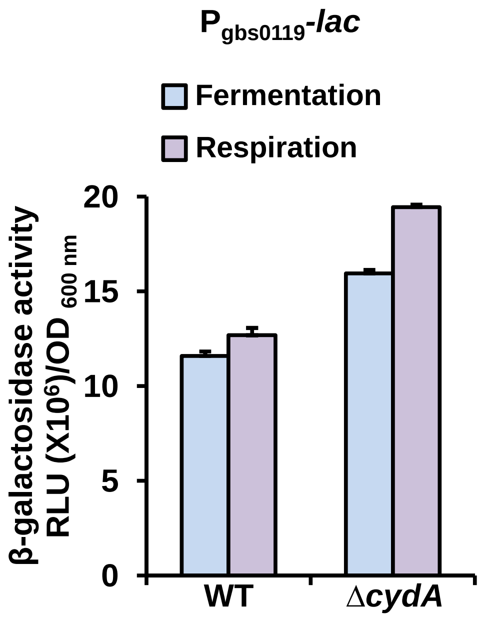
P gbs0119-lac
Fermentation
Respiration
β-galactosidase activity RLU (X10^6)/OD 600 nm
20
15
10
5
0
WT
ΔcydA

FIGURE S7

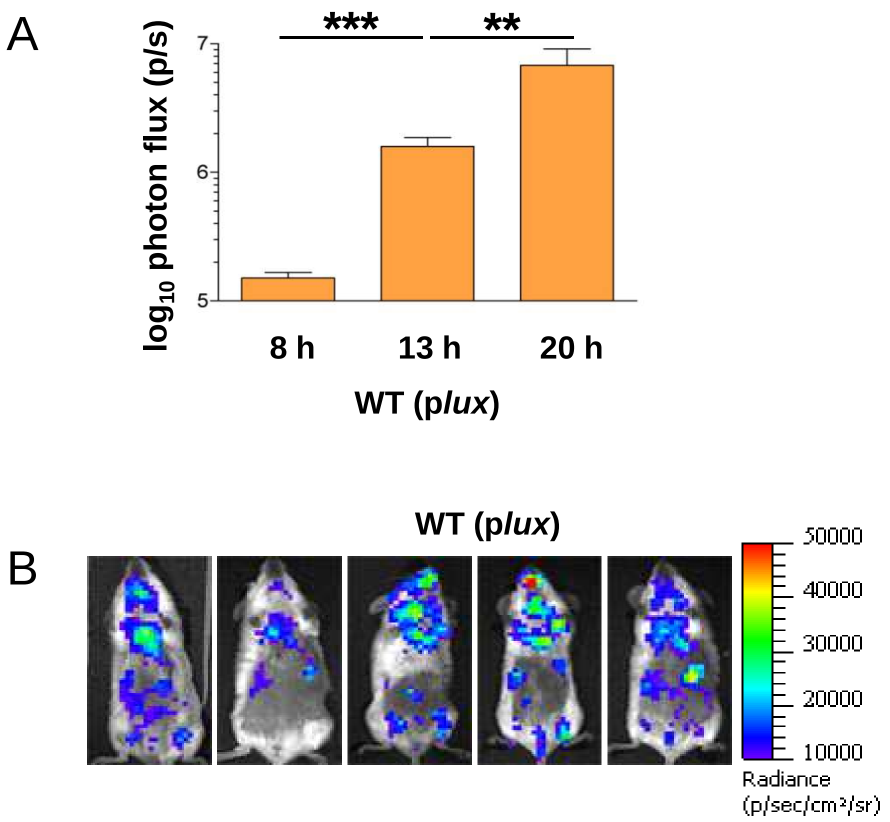
A
***
**
log10 photon flux (p/s)
7
6
5
8 h
13 h
20 h
WT (plux)
B
WT (plux)
50000
40000
30000
20000
10000
Radiance
(p/sec/cm²/sr)

FIGURE S8
